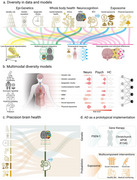# Implementing biomarkers in real‐world and diverse FTLD cohorts

**DOI:** 10.1002/alz70856_098814

**Published:** 2025-12-24

**Authors:** Agustin Ibanez

**Affiliations:** ^1^ Global Brain Health Institute (GBHI), University of California San Francisco (UCSF); & Trinity College Dublin, Dublin, Ireland; Latin American Brain Health Institute (BrainLat), Universidad Adolfo Ibañez, Santiago, Chile

## Abstract

Frontotemporal lobar degeneration (FTLD) presents unique challenges for diagnosis and intervention, particularly in diverse and underrepresented populations. This presentation focuses on the implementation of biomarkers in real‐world settings, emphasizing their integration into resource‐limited contexts and populations with heightened environmental and socioeconomic disparities. Traditional biomarker frameworks often neglect the complex interplay of whole‐body health and exposome factors—such as physical pollutants, social inequalities, and cultural determinants—that modulate disease onset and progression. In this talk, we introduce a multimodal diversity framework (Figure 1, Ibanez et al, Nature, under review) to biomarker application in FTLD, leveraging advanced neuroimaging, fluid biomarkers, and omics technologies while incorporating whole‐body health metrics (e.g., cardiometabolic conditions, inflammation) and exposome variables (e.g., air quality, socioeconomic status). By integrating these factors, we aim to enhance predictive accuracy and develop context‐sensitive diagnostic tools suitable for diverse global cohorts. Drawing from longitudinal data and collaborative efforts, this presentation outlines how biomarkers can reflect the broader biological embedding of exposome influences on brain health, particularly in low‐ and middle‐income regions. Validation studies demonstrate that biomarkers tailored to these contexts not only capture neurodegenerative patterns but also whol‐body healht and exposome influences. The findings underscore the potential of biomarkers as actionable tools for precision brain health, particularly when aligned with real‐world complexities. By bridging the gaps between laboratory innovation and practical application in diverse settings, this approach advocates for equitable access to diagnostic and therapeutic resources, addressing the global disparities in FTLD care.